# Supplementary material for: An effective protocol to isolate and mechanically test silk fibers spun by Osmia lignaria Say (Hymenoptera: Megachilidae) fifth instar larvae
Source: PLoS One. 2025 Feb 26;20(2):e0318918. doi: 10.1371/journal.pone.0318918 (PMC11864535; doi:10.1371/journal.pone.0318918)
Supplement: S1 File — https://doi.org/10.17504/protocols.io.bp2l6d8bkvqe/v1 (PDF) [file pone.0318918.s001.pdf]

# An effective protocol to isolate and mechanically test silk fibers spun by Osmia lignaria

O

Oran Wasserman

USU

**Collection Info:** Oran Wasserman: An effective protocol to isolate and mechanically test silk fibers spun by Osmia lignaria. **protocols.io**
**https://protocols.io/view/an-effective-protocol-to-isolate-and-mechanically-dvdg623w**

**Created:** December 13, 2024

**Last Modified:** December 17, 2024

**Collection Integer ID:** 115848

**Keywords:** X-ray film sheets , Silk-spinning larva , C-card , MTS machine

## Abstract

This collection of protocols describes the isolation and mechanical testing of *Osmia lignaria* silk fibers.

## Files

SEARCH

### Protocol

NAME

C-cards preparations

VERSION DVDD6226

CREATED BY

Oran Wasserman
USU

OPEN →

### Protocol

NAME

Fiber Isolation and Mounting onto C-cards

VERSION DVDE623E

CREATED BY

Oran Wasserman
USU

OPEN →

### Protocol

NAME

Fiber Diameter Measurement and Mechanical Testing

VERSION DVDF623N

CREATED BY

Oran Wasserman
USU

OPEN →
