# Supplementary material for: An effective protocol to isolate and mechanically test silk fibers spun by Osmia lignaria Say (Hymenoptera: Megachilidae) fifth instar larvae
Source: PLoS One. 2025 Feb 26;20(2):e0318918. doi: 10.1371/journal.pone.0318918 (PMC11864535; doi:10.1371/journal.pone.0318918)
Supplement: S2 File — https://doi.org/10.17504/protocols.io.ewov1d28pvr2/v1 (PDF) [file pone.0318918.s002.pdf]

C-cards preparations

In 1 collection

Oran Wasserman
USU

Protocol Info: Oran Wasserman: C-cards preparations. **protocols.io** **https://protocols.io/view/c-cards-preparations-dvdd6226**

Created: December 12, 2024

Last Modified: December 12, 2024

Protocol Integer ID: 115845

Keywords: X-ray film sheets , Mounting

Abstract

This protocol details the preparation of C-cards.

Materials

Materials:

- X-ray film sheets (Fuji Medical, 100NIF, 35 x 17 centimeters [cm])

Protocol

1 The outer layer of undeveloped X-ray film sheets is rinsed off using warm water and trimmed to 31 x 31 cm to fit the cutting mat (Silhouette CUT-MAT-12-3T).

2 The trimmed undeveloped X-ray film sheets are loaded into a Silhouette Cameo 4 equipped with a Type B AutoBlade (Silhouette SILH-BLADE-AUTO-2), set to 10, in the first blade slot and a 3 millimeter [mm] Kraft Blade (Silhouette SILH-BLADE-KRAFT-2) set to 15-20 in the second slot.

3 A repeating, custom design of the C-Cards is opened on the software (Silhouette Studio 4.5), which is then sent to the machine via a connecting USB cord to be cut with 2-4 passes with the first blade and 3-6 passes with the second blade, depending on blade sharpness.

4 The C-cards used are made with an 8 mm gap for testing and 9.5 mm mounting arms (Fig. S1).

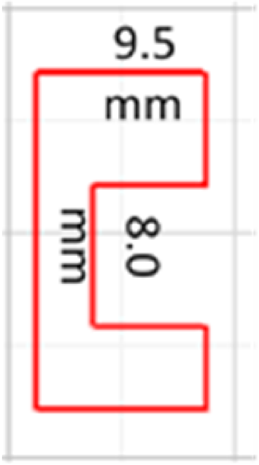

Figure S1. A single sample card design was used in Silhouette Cameo.
