# Supplementary material for: An effective protocol to isolate and mechanically test silk fibers spun by Osmia lignaria Say (Hymenoptera: Megachilidae) fifth instar larvae
Source: PLoS One. 2025 Feb 26;20(2):e0318918. doi: 10.1371/journal.pone.0318918 (PMC11864535; doi:10.1371/journal.pone.0318918)
Supplement: S3 File — https://doi.org/10.17504/protocols.io.x54v9r7mqv3e/v1 (PDF) [file pone.0318918.s003.pdf]

Fiber Isolation and Mounting onto C-cards

In 1 collection

O
Oran Wasserman
USU

Protocol Info: Oran Wasserman: Fiber Isolation and Mounting onto C-cards. **protocols.io** **https://protocols.io/view/fiber-isolation-and-mounting-onto-c-cards-dvde623e**

Created: December 12, 2024

Last Modified: December 17, 2024

Protocol Integer ID: 115846

Keywords: Fiber isolation , Magnification, C-card

Abstract

This protocol details the isolation of fiber and its mounting onto cards.

Materials

Materials:

- Black glass mat
- Dissection microscope
- Dissection forceps
- Dissection scissors
- Microscope slide
- Single-edge razor blade
- Super glue
- Transparent tape
- Wood toothpick 10.16 cm
- X-ray film sheets C-cards

Before start

Note

It is important to check larval development under the dissection microscope regularly. It is preferable to perform fiber isolation within the first few hours after the larva begins to spin silk. Larvae can spin silk at the bottom of the well, which requires higher total magnification past 20X.

Protocol

30m

1 Using scissors, make appropriate cuts from the apex to the body of the silk mesh, bend back the existing silk, and provide better access to the larva.

> **Note**
>
> In some cases, it may be appropriate to use forceps to pull off excess silk from the straw liner without disturbing the larva.

2 Position the well containing the silk-spinning larva under a dissecting microscope at 20X total magnification.

3 **Isolate fibers using one of two techniques (S1 Video):**

3.1 Use forceps to pinch the lengthening fiber as the larva pulls the fiber from one wall connection to another. Gently pull the fiber at a similar speed at which the larva is moving its head to the other wall, directing the larva to pull away from the forceps instead of from the well wall due to the equal and opposite force.

> **Note**
>
> Excess stretching can change the diameter of the isolated fiber, resulting in altered mechanical properties of isolated silk fibers.

3.2 As the larva extrudes a new strand, position the forceps in front of the larva or gently touch the forceps to the mouth or head of the larva to encourage attachment of the beginning fiber to the forceps. Follow the same method of pulling the extruded silk away from the larva at a speed matching the larva. If done correctly, a fiber of approximately 15 cm in length, on average, can be collected before the fiber strand breaks.

4 Transfer the isolated fiber to a black glass mat for visualization during the carding process (S2 Video and S3 Video depict steps 4-15).

Note

The fibers are small and typically have white pigmentation. Handling the fibers under a dark surface will aid in the carding process.

5 Using pre-cut approximately 2 x 5 mm pieces of clear tape and forceps. Apply tape perpendicularly to both ends of the fiber.

6 Using pre-cut 4 x 5 mm pieces of tape, apply tape approximately 12 mm from the end tape, perpendicular to the fiber, leaving enough room for silk to be seen between the piece of tape and the beginning of the gap of the C-card.

7 While the fiber is still on the glass mat, use a razor blade to cut down the middle of the widest portion of the tape.

8 Position one end of a C-card alongside the fiber between the two pieces of tape using the smaller isolated fiber.

9 Using a pair of forceps, lift one piece of tape and gently place it at the end of the C-card between the two pieces of tape, leaving room to glue the fiber to the card.

Note

This ensures that you will keep the fiber slack and not apply unnecessary stress or strain to the fiber.

10 Press down the piece of tape using a rod or another pair of forceps, allowing the first pair of forceps to be released from the tape without pulling on the fiber.

11 Repeat Steps 9-10 for the other end of the fiber, ensuring that the fiber is taut across the gap of the card when mounted to the C-card.

Note

Keep the fiber slack while handling to avoid applying unnecessary stress or strain to the fiber.

12 Transfer and secure the C-card onto a microscope slide using pieces of tape.

Note

Doing this allows for the safe transfer and organization of the fibers.

13 Secure the fiber onto the C-card using super glue. Pour superglue into a small container and apply a small amount to each end of the fiber near the notch/gap of the card using a wood toothpick.

Note

Applying super glue to the fiber directly will secure the fiber to the C-card and provide higher consistency in the mechanical tests.

14 Allow the glue to dry for at least 00:30:00 before mechanical testing. 30m

15 Repeat steps 8-14 for the fiber that was transferred to the black mat.

Note

Once the transferred fiber has been utilized for testing, isolate a new fiber and repeat steps 5-14.
