# Supplementary material for: An effective protocol to isolate and mechanically test silk fibers spun by Osmia lignaria Say (Hymenoptera: Megachilidae) fifth instar larvae
Source: PLoS One. 2025 Feb 26;20(2):e0318918. doi: 10.1371/journal.pone.0318918 (PMC11864535; doi:10.1371/journal.pone.0318918)
Supplement: S4 File — https://doi.org/10.17504/protocols.io.eq2ly6x3mgx9/v1 (PDF) [file pone.0318918.s004.pdf]

Fiber Diameter Measurement and Mechanical Testing

In 1 collection

Oran Wasserman
USU

Protocol Info: Oran Wasserman: Fiber Diameter Measurement and Mechanical Testing. **protocols.io** **https://protocols.io/view/fiber-diameter-measurement-and-mechanical-testing-dvdf623n**

Created: December 13, 2024

Last Modified: December 13, 2024

Protocol Integer ID: 115847

Keywords: MTS machine , C-card, Fiber

Abstract

This protocol details the measurement and mechanical testing of fiber.

Materials

Materials:

- Motic BA310 microscope and the Motic Image Plus 3.1 program (Schertz, Texas, USA)
- Single-edge razor blade
- Dissecting scissors
- Tensile testing equipment (Material Testing System Synergie 100 tensile testing instrument with a custom 10 gram [g] load cell [Transducer Techniques, Newark, California, USA])

Protocol

1. Place the fiber under a Motic BA310 microscope with the Motic Image Plus 3.1 program and take diameter measurements for each fiber at a minimum of 400X total magnification.

2. Focus on the fiber at three points along the fiber (both ends and middle of the fiber) and, using the software, capture the screen at each location. Using the ruler function in the software, take the diameter at three different points in each captured picture. The resulting nine measurements are to be averaged and recorded.

3. Remove the C-card from the microscope slide using a razor blade to cut the tape securing the C-card to the slide.

4. Load the fiber into the MTS machine by inserting the C-card into the upper clamp and tightening it so the clamp rests on the tape, super glue, and C-card.

5. Lower the MTS machine to the lower clamp on the 10 g load cell and close the clamp in the same way as the upper clamp.

6. Lower the machine further so that the C-card begins to bow and the fiber has slack, allowing the C-card to be cut without imposing stress on the fiber.

7. While holding the C-card, cut the length of the C-card with scissors to prevent any spring effect from straining the fiber.

8. Raise the MTS machine until the fiber is almost taut, but not fully taut, to avoid imposing strain on the fiber.

9. Run the MTS machine, a pull rate of five millimeter per minute [mm/min] and a data collection rate of 120 hertz [Hz] were used.

10. Export the data and analyze it in Microsoft Excel to calculate each fiber's maximum tensile stress, maximum strain, elastic modulus, and toughness.
