# Supplementary material for: An effective protocol to isolate and mechanically test silk fibers spun by Osmia lignaria Say (Hymenoptera: Megachilidae) fifth instar larvae
Source: PLoS One. 2025 Feb 26;20(2):e0318918. doi: 10.1371/journal.pone.0318918 (PMC11864535; doi:10.1371/journal.pone.0318918)
Supplement: S6 File — (DOCX) [file pone.0318918.s006.docx]

An effective protocol to isolate and mechanically test silk fibers spun by *Osmia lignaria* Say (Hymenoptera: Megachilidae) fifth instar larvae

**S6 File:** Guide for mechanical properties analysis

The raw data obtained from the tensile testing of fibers using the MTS software TestWork 4 should be exported as a .txt file. This data can then be imported into a pre-formatted Excel template, where the fiber diameters have been previously recorded. Ensure that the appropriate delimiter (“comma”) is selected to correctly separate the data into columns during the import process. To analyze the data, set up the following key formulas in an Excel spreadsheet:

**Zeroing out Extension**

$$L_{actual}=L_{at point}- L_{starting value}$$

Where L_starting value_ is the point where the data for the test starts being saved (data is logged for a time before starting; in Excel, this point needs to be locked so it is the same throughout). The L_at point_ is the current extension value. The extension is in mm.

**Zeroing out Force**

$${F_{actual}=F}_{at point}- F_{starting value}$$

Where F_starting value_ is the point where the data for the test starts being saved (data is logged for a time before starting, in Excel this point needs to be locked so it is the same throughout each test). F_at point_ is the current force value. The force is in Newtons (N).


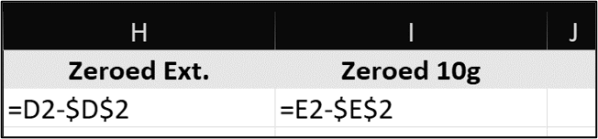


**Figure S6-1.** An example from our Excel file for zeroing out extension and force. In this example, the D column contains the raw extension values, and the E column contains the raw load values. The H and I columns contain the zeroed-out forms of those data values.

**Stress (σ)**

$$\sigma=\frac{F}{A}$$

Where F is the force applied (calculated above in the “Zeroing out Force” equation), and A is the cross-sectional area of the fiber, in square millimeters (mm^2^). The final units are N/mm^2^ or megapascals (MPa).

**Strain (ε)**

$$\varepsilon=\frac{\Delta L}{L_{0}}$$

Where ΔL is the change in length (calculated above in the “Zeroing out Extension” equation), and L_0_ is the original gauge length of the fiber.


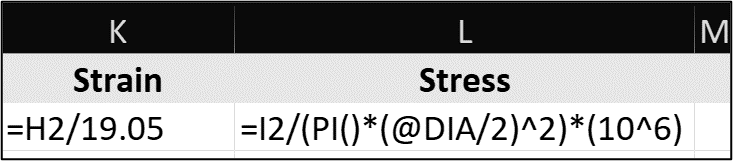


**Figure S6-2.** An example of strain and stress is from our Excel file. Strain is the gap (mm) of the length of the fiber. The denominator for stress is the area of the circle (fiber shape) converted to mm^2^.

**Elastic Modulus (E)**

$$E=\frac{\Delta\sigma}{\Delta\varepsilon}$$

Where Δσ is the change in stress, and Δε is the change in strain within the linear elastic region of the stress-strain curve. Use the slope function in Excel to calculate the elastic modulus, ensuring consistent data points are selected across all fibers tested. When using the MPa stress (Y-value) and the mm/mm strain (X-value), you get units of MPa, which can get quite large, so they are often converted to gigapascals (GPa) for readability.

**Toughness (U_T_)**

$$U_{T}= Area underneath the stress-strain \left( \sigma-\varepsilon\right) curve=\int0^{\varepsilon_{f}}\sigma d\varepsilon$$

To calculate toughness, integrate the stress-strain curve from zero strain to the strain at failure (ε_f_) using numerical integration techniques, such as the trapezoidal rule.

**Trapezoidal Rule**

$$U_{T}\approx\Delta\varepsilon\times(0.5\times\sigma_{0}+\sigma_{1}+\sigma_{2}+\ldots+\sigma_{n-1}+0.5\times\sigma_{n})$$

Use the appropriate unit for toughness (also called energy to break), typically megajoules per meter-cubed (MJ/m^3^) or joules per meter-cubed (J/m^3^).


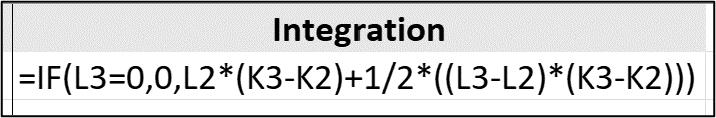


**Figure S6-3.** An example from our Excel file of integration calculations includes an IF() statement that starts at 0. The final value for toughness is the sum of all the calculated values.

**Running Analyses and Calculations**

1. After loading the data into Excel, enter the equations as they fit your sheet design.
   1. This may differ from the examples given in Figures S6-1, S6-2, and S6-3.
2. Select the starting point.
   1. This is typically after the raw extension data is no longer zero or negative and when the load values are increasing without drastic drops.
      1. If there is noise in the data, this point is more difficult to spot.
      2. The data can contain noise depending on the sample and instrument used. A 5-point moving average can reduce the noise and be applied to the stress values.
      3. Place/paste in your zeroing extension, zeroing force, strain, stress, and toughness equations at this row.
3. Adjust the formulas for [zeroing the extension](#_Zeroing_out_Extension:) and [load columns](#_Zeroing_out_Force:) to subtract the initial value from itself, ensuring these columns start at zero.
4. Modify the [stress formula](#_Stress_(σ):) to use the correct fiber diameter measured by the microscope (micron).
5. Modify the [strain formula](#_Strain_(ε):) to use the correct gauge length (mm).
   1. The gauge length only changes if you change the length of fiber you are testing.
6. Drag the zeroing extension, zeroing force, strain, stress, and toughness equations down to calculate the full dataset for that particular sample.
   1. Backtrack and clear values were gathered after the sample broke.
   2. Or drag until you reach the point where the load is maximized or does not drop, then climb back up (occasionally not the same value).
7. Copy and paste the stress and strain columns as links into a separate tab to generate a stress-strain curve for each fiber.
   1. Ensure you paste them in the same order: Strain first, then Stress.
   2. Tensile stress can be found to be the maximum stress on this curve.
      1. In Excel, use the max() function for the maximum stress (MPa) and the maximum strain (mm/mm).
   3. Look at the graph and determine the [linear elastic region](#_Elastic_Modulus_(E):) (near the beginning), then use the slope() function in Excel to determine the slope of that region.
      1. X-values are strain, and Y-values are stress (they are entered Y then X).
      2. If the value is too large and not readable, convert them to GPa (divide the slope value by 1000). This is the elastic modulus.
8. Copy and paste the [integration column](#_Toughness_(U_T):) as a link into a separate tab.
   1. Sum up the entire contents of that column to get the toughness.
9. Repeat this data analysis process for each fiber tested to generate a comprehensive set of mechanical properties.
10. Create a summary table combining the diameter, strain, stress, toughness, and elastic modulus values for each sample of the same treatment group.
    1. Ensure all units are consistent.
    2. Include all individual sample measurements.
11. Calculate statistical measures for each mechanical property within treatment groups.
    1. Use the AVERAGE() function in Excel to determine mean values.
    2. Use the STDEV.S() function in Excel to calculate standard deviations.
    3. Include these summary statistics at the bottom of the data table.
