# Supplementary material for: An effective protocol to isolate and mechanically test silk fibers spun by Osmia lignaria Say (Hymenoptera: Megachilidae) fifth instar larvae
Source: PLoS One. 2025 Feb 26;20(2):e0318918. doi: 10.1371/journal.pone.0318918 (PMC11864535; doi:10.1371/journal.pone.0318918)
Supplement: Attachment — Submitted filename: Response to Reviewers.docx [file pone.0318918.s012.docx]

**Authors remarks**: We appreciate the care and time the editor and reviewers have taken in their comments on our manuscript. Below, we provide a detailed response to all the comments assigned to the manuscript. If any additional part needs to be revised, please let us know, and we will be happy to provide an explanation or a revision.

**REVIEWER 1’S COMMENTS TO THE AUTHORS**

Comments:
The protocol for isolation and mechanical testing of silk fibers spun by the blue orchard bee, an important pollinator of orchards in North America, offers a minimally invasive approach that allows larvae to proceed with cocoon formation after fiber isolation. This protocol allows larval rearing of this ecological important insects and thus, it is very important for investigating the relationship between silk biochemistry and mechanical properties without killing the bees. In addition, the presented protocol can also be used for testing of silk from other solitary bee species. Therefore, the relevance of this protocol is very high. The protocol is very detailed and easy to follow because of the very detailed description of the single steps. In addition, the discussion of possible limitations is very helpful to avoid errors in the implementation of the protocol by other scientists at an early stage.

| **REVIEWER 2’S COMMENTS TO THE AUTHORS** | |
| --- | --- |
| Comments:  The authors needs to answer certain queries provided which can be included in the manuscript: | |
| **Specific points from Reviewer 2:** | **Author’s remarks:** |
| 1. What are the alternative materials that can replace 3-D printed well plates for rearing Osmia lignaria larvae? | While we did not directly test alternative rearing apparatuses, in theory, any rearing apparatus that can hold the straw liner (e.g., 48-well culture plates) can be used. Plates do not necessarily have to be 3-D printed; we had them available from other projects. Another option, making a cast from a 3-D-printed plate, is provided in Williams et al. 2024. |
| 2. How does the timing of fiber isolation affect the mechanical properties of the silk? | In our Lab Protocol manuscript, we haven’t investigated how the timing of fiber isolation affects the mechanical properties of silk. Future studies would benefit from examining if and how the timing of isolation influences mechanical properties*.* In line 258, testing parameters also refer to fiber isolation and testing timing. We appreciate this important question and have added a discussion of this as a key parameter for future studies in the revised manuscript. |
| 3. What are the optimal environmental conditions (temperature, humidity) for silk spinning in *Osmia lignaria*? | In our protocol, we have not investigated the optimal environmental conditions for silk spinning in *Osmia lignaria*. Future investigations could examine how temperature and humidity affect the *O. lignaria* spinning process, including both the properties and quantity of silk produced. We have added sentences to the manuscript (lines 250-252 and 259) discussing this important question. |
| 4. How can the isolation method be adapted for smaller bee species with different cocoon construction methods? | The main adaptation needed for smaller bee species is adjusting the well diameters during development. We discuss this adaptation to the protocol in lines 267-270 of the manuscript. |
| 5. Does the method of cutting the silk mesh impact the tensile strength or elasticity of the isolated fibers? | In our method, we cut the silk mesh only to access the larvae. We do not test fibers from the mesh itself due to their fragility and fusion, as described in lines 213-220 of the manuscript. |
| 6. How do variations in fiber diameter correlate with mechanical properties like tensile strength and elasticity? | We observed variations *in O. lignaria* silk, where fiber diameters ranged from 1.9 to 46.4 μm. This variation in diameter contributes to the observed variability in mechanical properties, as the diameter is included in calculations to determine stress, toughness, and elastic modulus. |
| 7. Can the tensile testing protocol be adapted for higher-throughput analyses of multiple fibers simultaneously? | While technically possible, we haven't investigated high-throughput methods in our protocol. Given that there are many variables during the isolation and testing of *O. lignaria silk fibers'* mechanical properties, extensive research would be needed to establish reliable high-throughput analysis methods of multiple fibers simultaneously. |
| 8. What are the effects of different glues on the mechanical properties of silk during C-card mounting? | We haven't investigated how different glues affect the mechanical properties during C-card mounting in our protocol. Future studies with larger datasets will be needed to understand how mounting parameters, including glue selection, influence silk fiber mechanical properties. |
| 9. How does the choice of testing environment (e.g., ambient vs. controlled humidity) affect mechanical test outcomes? | We haven't investigated how testing environment conditions affect mechanical properties in our protocol. While previous studies with other silks have shown that environmental factors can influence mechanical properties, determining specific effects of temperature and humidity on *O. lignaria* silk will require future research. |
| 10. What molecular markers in the silk indicate the health and nutritional status of the larvae? | We haven't investigated molecular markers in the silk developing our isolation protocol, in our study. Future studies focusing on characterizing the nutritional components of the provisions and performing feeding assays could establish a correlation between the health and nutritional status to the mechanical structural properties of the silk fibers. |
| 11. How does the chemical composition of Osmia lignaria silk compare to that of other bee or silkworm species? | Future studies can use our protocol to isolate *O. lignaria* silk fibers, which could then be analyzed using various analytical techniques like LC-MS/MS to determine their chemical composition in comparison to other bee or silkworm species. |
| 12. Can the silk from Osmia lignaria be used as a biomarker for exposure to environmental stressors, such as pesticides? | Once the fibers are isolated using our protocol, they can be subjected to various analytical tests. Future studies involving feeding or exposure assays would be needed to determine how *O. lignaria* silk could serve as a biomarker for environmental stressors such as pesticides. |
| 13. What are the biodegradation properties of Osmia lignaria silk in various environmental conditions? | We haven't investigated biodegradation properties in our protocol. Once the fibers are isolated, they could be subjected to controlled environmental exposure studies to determine their degradation rates and patterns under various conditions. However, this type of analysis would require additional research. |
| 14. How do the mechanical properties of silk from solitary bees compare with those of social bees, such as honeybees? | Our protocol provides a foundation for isolating and testing silk fibers from *O. lignaria*. While we haven't conducted comparative analyses, this method could be adapted to study the mechanical properties of silk from both solitary and social bee species in future research. |
| 15. What are the evolutionary implications of the observed mechanical differences between Osmia lignaria silk and other insect silks? | Our protocol focuses specifically on the isolation and mechanical testing of *O. lignaria* silk fibers. Understanding the evolutionary implications of mechanical differences between *O. lignaria* silk and other insect silks would require additional comparative studies across species, which is not part of the scope of our protocol. |
| 16. Are there phylogenetic correlations between the mechanical properties of bee silks and their nesting behaviors? | While our protocol enables the isolation and testing of silk fibers from *O. lignaria* cocoons, we haven't investigated phylogenetic relationships. Understanding correlations between silk mechanical properties and nesting behaviors across bee species would require comprehensive studies of cocoon structure and function across multiple species. |
| 17. What improvements can be made to reduce the variability in fiber diameter during isolation? | That is a great question. Diameter variations in both native and recombinant silk have been reported previously (Greco et al., 2022). To answer that question, a future investigation is required to optimize an isolation technique that focuses on reducing variability in fiber diameter. |
| 18. How does the larval diet during development influence the quality and mechanical properties of the silk? | Our protocol hasn't investigated how larval diet influences silk's mechanical properties. However, the method we developed for silk isolation and testing could be used in future feeding assays to examine relationships between larval nutrition and silk properties. |
| 19. Can the protocol be modified to allow for automated fiber isolation? | We haven't investigated automating fiber isolation in our protocol. While automated collection methods like mechanical spools have been used with other silk-producing species, they would need to be validated against our manual method, which matches larval head movements to ensure fibers aren't artificially stretched during collection. |
| 20. What are the potential industrial applications of silk isolated using this protocol (e.g., biomedical materials, eco-friendly textiles)? | Our protocol focuses on isolating silk for research purposes rather than industrial applications. While *O. lignaria* silk could potentially be used for biomedical applications, this would require extensive research. Utilizing *O. lignaria* silk for industrial applications may be more feasible through the recombinant production of silk proteins. |
